# Promyelocytic leukemia protein regulates angiogenesis and epithelial–mesenchymal transition to limit metastasis in MDA‐MB‐231 breast cancer cells

**DOI:** 10.1002/1878-0261.13501

**Published:** 2023-09-04

**Authors:** Amalia P. Vogiatzoglou, Syrago Spanou, Nikoleta Sachini, Elias Drakos, Christoforos Nikolaou, Takis Makatounakis, Androniki Kretsovali, Joseph Papamatheakis

**Affiliations:** ^1^ Institute of Molecular Biology and Biotechnology (IMBB) Foundation for Research and Technology‐Hellas (FORTH) Crete Greece; ^2^ Department of Biology University of Crete Heraklion Greece; ^3^ ADC Therapeutics Limited London UK; ^4^ Department of Pathology, Medical School University of Crete Greece; ^5^ Biomedical Sciences Research Center “Alexander Fleming” Institute for Bioinnovation Vari Greece

**Keywords:** angiogenesis, breast cancer, EMT, metastasis, PML, transcriptomics

## Abstract

Promyelocytic leukemia protein (PML) modulates diverse cell functions that contribute to both tumor suppressor and pro‐oncogenic effects, depending on the cellular context. We show here that *PML* knockdown (KD) in MDA‐MB‐231, but not MCF7, breast cancer cells, prolonged stem‐cell‐like survival, and increased cell proliferation and migration, which is in line with gene‐enrichment results from their RNA sequencing analysis. Of note, increased migration was accompanied by higher levels of the epithelial–mesenchymal transition (EMT) regulator Twist‐related protein 2 (TWIST2). We showed here that PML binds to TWIST2 via its basic helix–loop–helix (bHLH) region and functionally interferes with the suppression of the epithelial target of TWIST2, CD24. In addition, PML ablation in MDA‐MB‐231 cells led to higher protein levels of hypoxia‐inducible factor 1‐alpha (HIF1a), resulting in a higher cell hypoxic response. Functionally, PML directly suppressed the induction of the HIF1a target gene vascular endothelial growth factor A (VEGFa). In line with these results, tumor xenografts of MDA‐MB‐231 PML‐KD cells had enhanced aggressive properties, including higher microvessel density, faster local growth, and higher metastatic ability, with a preference for lung. Collectively, PML suppresses the cancer aggressive behavior by multiple mechanisms that impede both the HIF–hypoxia–angiogenic and EMT pathways.

## Graphical abstract

Promyelocytic leukemia protein (PML) is an essential factor for the assembly of nuclear bodies (PML‐NBs), which regulate diverse cellular processes. Here, we show that PML loss increases *in vitro* cancer cell proliferation and migration. In addition, tumor xenografts have a faster local growth and higher metastatic ability. We ascribe these effects to the inhibitory role of PML on both the hypoxia–HIF–angiogenic axis and the pro‐migratory action of epithelial–mesenchymal transition factors such as TWIST2.

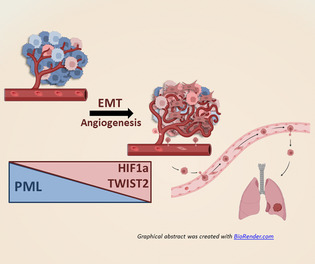

**Abbreviations**
APL: acute promyelocytic leukemia
bHLH: basic helix–loop–helix protein structural motif
CLD: claudin
EMT: epithelial–mesenchymal transition
FSCN1: fascin actin‐bundling protein 1
HIF1a: hypoxia‐inducible factor 1‐alpha
ID1: inhibitor of DNA binding 1
PML: promyelocytic leukemia protein
PML‐NBs: PML nuclear bodies
RAR‐α: retinoic acid receptor alpha
RBCC: ring‐B box‐coiled coil
SLUG: snail family transcriptional repressor 2
SNAIL1: snail family transcriptional repressor 1
TNBC: triple‐negative breast cancer
TWIST2: twist‐related protein 2
VEGFa: vascular endothelial growth factor A

## 1 Introduction

Breast cancer is characterized by genetic and epigenetic complexity and phenotypic heterogeneity [1]. Differences between breast cancer subtypes or patients referred to as intertumoral heterogeneity as well as cellular diversity within the same tumors [2] are dynamically driven by genetic and epigenetic evolution and have an impact in patient's prognosis and therapy. Major current efforts are directed toward the elucidation of factors that modify the action of critical oncogene or tumor suppressor pathways that regulate invasiveness, metastatic potential, and drug response with predictive or therapeutic importance.

The promyelocytic leukemia (PML) gene was first described in the early 1990s at the point of chromosomal translocation t (15, 17), where it was found to encode an oncogenic chimeric protein emerging from the fusion of PML and the retinoic acid receptor alpha (RAR‐α) in patients with acute promyelocytic leukemia (APL). The PML‐RAR chimeric protein prevents the differentiation of bone marrow progenitor cells, sustaining their self‐renewal capacity and preventing their apoptotic process, leading to leukemogenesis [3, 4]. Promyelocytic leukemia protein is the key component of PML‐NBs, which are nuclear membrane‐less compartments self‐assembled through the RBCC (Ring‐B box‐coiled coil) motif interactions. By interacting and/or participating in the post‐translational modifications of various nuclear proteins, PML‐NBs regulate a variety of cellular processes such as apoptosis, proteolysis, cellular aging, cell self‐renewal capacity, DNA damage response, telomere stability, gene expression, and chromatin/epigenetic states [5, 6]. For example, PML is highly expressed during murine mammary gland development, it is barely detectable during pregnancy and lactation, and it is necessary for lineage commitment of bi‐potent luminal progenitor cells [7]. Moreover, PML is essential for sustaining naïve pluripotent state in mouse embryonic stem cells [8]. The role of PML in neoplasias seems to be complex. PML protein is lost in human cancers of various histologic origins, including breast cancer [9]. Various reports support a tumor suppressive role for PML [10]. Using an inducible expression of PML IV in the triple‐negative breast cancer (TNBC) Claudin (CLD) low subtype MDA‐MB‐231 cell line, we found strong inhibition of cell proliferation and tumor sphere formation in a reversible manner [11]. Other studies point to a pro‐survival role of PML in nontumorigenic or tumorigenic breast cell lines and find that high PML levels correlate with poor prognosis in a breast cancer cohort [12]. Similarly, high PML expression in chronic myeloid leukemia maintains hematopoietic stem cells and PML ablation leads to an increase in their initial cycling activity that eventually leads to their eradication [13]. These studies suggest that PML may have divergent cell effects not only in different cell contexts but also in different temporal windows, that is, short‐ vs long‐term effects.

Here, we show that PML loss enhances cell migration *in vitro* and metastatic ability *in vivo* of TNBC cells. Cells derived from *in vivo* primary tumors or lung metastasis stably maintain an *in vivo* aggressive phenotype upon regrafting and transcriptionally resemble the earlier described lung metastasis signature (LMS) [14]. We attribute the above to at least two independent functions of PML: First, we found that PML loss promotes the hypoxia–HIF signaling and tumor xenograft angiogenesis. Second, PML physically interacts and functionally affects the activity of various bHLH or Zinc finger type EMT mediators, such as TWIST2, in line with transcriptional enrichment in adhesion‐related genes. Thus, PML acts as a tumor suppressor by limiting tumor angiogenesis and EMT.

## 2 Materials and methods

### 2.1 Plasmids, DNA, and shRNA transfections

Plasmids with PMLIV, PMLIII, and PMLI isoforms have been previously described [15]. For immunoprecipitation and localization experiments, the PMLIV, PMLIII, and PMLI isoforms were fused to the mRED vector (Clontech, Mountain View, CA, USA). TWIST1, TWIST2, SNAIL1 (Addgene #16225 [16]), and SLUG (Addgene #25696) were fused to GFP‐C (Clontech). mCherry LEGO‐C2 was provided by K. Weber. The 5xHREVEGFLuc reporter was provided by S. Simos. The CD24‐Luc was constructed by cloning the −1700 to +79 bp (PCR amplified using CD24 forward 5′‐GAGCTAAAGTGACTGACCTTGAAGGCACAA‐3′ and reverse 5′‐CGTCTAGCAGGATGCTGGGTGCTTG‐3′ primers) of the CD24 promoter upstream of the pGL3luc reporter. Transient transfections were performed using the calcium phosphate method or Lipofectamine 2000 (Thermo Fisher Scientific, Waltham, MA, USA) according to the manufacturer's instructions. The lentivirus production and infection protocol have been previously described in detail [17].

### 2.2 Cell culture and generation of stable cell lines

Human breast cancer cell lines MDA‐MB‐231 (RRID:CVCL_0062) and HEK293T (RRID:CVCL_0063) were provided from ATCC and MCF7 (RRID:CVCL_0031) from DSMZ and cultured in DMEM, 10% FBS and gentamycin 1%, 5% CO_2_ and 37 °C. Cell lines were authenticated by STR analysis cell and were periodically screened for being mycoplasma negative. For the generation of short‐hairpin of stable, knockdown cell lines, cells were infected by puromycin or G418 resistance pLKO.1 (Addgene #8453, #13425) lentiviral vectors carrying the relevant shRNAs followed by drug selection. The shPML RNA target sequences were as follows: Sh0:5′‐AGATGCAGCTGTATCCAAG‐3′ [18], Sh1: 5′‐GCTGTATCCAAGAAAGCCA‐3′, and sh2: 5′‐CCAACAACATCTTCTGCTCC‐3′. The shHIF1α sequence was: 5′‐UGAGGAAGUACCAUUAUAU‐3′ [19]. Knockdown (KD) was evaluated by western blot and/or mRNA analysis using suitable primers.

### 2.3 Tumor sphere forming assay

For tumor sphere‐forming assays, cells were cultured in DMEM‐F12 1 : 1 (Gibco, Waltham, MA, USA) containing B27 (1 : 50), bFGF (20 ng·mL^−1^), EGF (20 ng·mL^−1^), and 0.2% methylcellulose at 1000 cells·mL^−1^ using in ultra‐low attachment plates. After 8 days, spheres of ≥ 70 μm size were enumerated and, if required, were dispersed by accutase and cells were counted for next passage.

### 2.4 RNA extraction and qRT‐PCR

Total RNA extraction was performed using Nucleozol (Macherey‐Nagel, Düren, Germany). Next, 2 μg RNA was used to generate library cDNAs using the enzyme M‐MuLV Reverse Transcriptase (New England, Biolabs, USA) together with an RNase inhibitor (New England, Biolabs, Ipswich, MA, USA) according to the manufacturer's protocol. The relative abundance of each gene transcript was measured by qRT‐PCR using the dye SYBR Green I (Invitrogen, Waltham, MA, USA). Relative mRNA expression was calculated after normalization against β‐actin or GAPDH levels. Set of primers used for qRT‐PCR are listed in Table S1.

### 2.5 RNA sequencing

Total RNA was isolated from control cells and PML‐KD using TRIzol (Invitrogen). For the MDA‐MB‐231 samples, the mRNA 3′‐UTR sequence method was used to create libraries with the QuantSeq 3′mRNA‐Seq Library Prep Kit (Lexogen, Vienna, Austria) for Ion Torrent‐Cat #012. And the sequencing was done with Ion S5, with Ion 540 Reagents. NEBNext Ultra II Directional RNA Library Prep Kit (New England Biolabs, Ipswich, MA, USA) for Illumina‐E7760 and NEBNext Poly (A) mRNA Magnetic Isolation Module‐E7490 were used for libraries for complete RNA sequencing of MCF7 cells. Sequencing was performed on the NextSeq 500 Illumina with FlowCell High 1x75 (IET, San Diego, CA, USA). hisat2 version 2.1 (genome mapper, Johns Hopkins University, Baltimore, MD, USA).

### 2.6 Analysis of differentially expressed genes

Differential gene expression analysis was performed with μetaseqr. The functional analysis was performed using the web tool gprofiler [20] and metascape (Bioconductor, https://bioconductor.org/) [21].

### 2.7 Western blot analysis (WB)

WB was done essentially as described by Sachini et al. [11]. In brief, whole‐cell lysates were prepared using RIPA cell lysis buffer (25 mm Tris pH 7.6, 150 mm NaCl, 1% NP‐40, 1% deoxycholate, 0.1% SDS, 1 mL PMSF) containing protease inhibitor cocktail (Complete; Sigma‐Aldrich Chemie GmbH, Taufkirchen, Germany), and protein concentration was determined by Bradford assay. Equal amounts of cell lysates were subjected to SDS/PAGE, followed by immunoblotting. The primary antibodies used for WB are listed in Table S2.

### 2.8 Protein immunoprecipitation (Immunoprecipitation‐IP)

Immunoprecipitation of protein complexes was performed as described earlier [11], using HEK293T cell extracts in RIPA lysis buffer following overexpression of specific proteins as described above. Two hundred micrograms of protein extracts was incubated with primary antibody overnight at 4 °C. The following day, 20 μL of protein G beads was added to each sample after washing with IP buffer (25 mm Tris/HCl pH 7.6, 150 mm NaCl), and reactions were incubated at 4 °C for three additional hours. Nonspecific proteins were washed away three times with NETN buffer (10 mm Tris/HCl pH 8.0, 250 mm NaCl, 5 mm EDTA, 0.5% NP‐40, 1 mm PMSF). SDS sample buffer was added, and the samples were boiled prior to SDS/PAGE analysis. Input lanes represent 10% of the lysate used for the IP.

### 2.9 FAC analysis

Flow cytometry was used to evaluate CD24 and CD44 surface expression (FACSCalibur by Becton Dickinson (BD; Franklin Lakes, NJ, USA). The cells were detached with trypsin–EDTA, and following centrifugation was suspended in PBS‐2% FCS‐0.1% NaN_3_ with specific antibodies of isotype controls.

### 2.10 Cell migration assay

For migration assays, cells were seeded into Millicell, Cell culture 8 μm inserts (Merck KGaA, Germany) in FBS‐free medium overlaying the lower compartment filled with FBs‐containing media. The next day, the upper side cells were scraped away and the insert was fixed with 70% ethanol, stained with crystal violet (Merck, Darmstadt, Germany), and counted under a microscope. The percentage of cells that have passed through the membrane in relation to the total number of cells seeded on the transwell is indicative of the migrating capacity of the cells.

### 2.11 Immunostaining and microscopy

Cells for immunostaining were cultured on 8‐well chamber, removable (80841, Ibidi GmbH, Lochhamer Schlag, Germany), and fixed in buffered 4% PFA/1XPBS for 5 min at room temperature, permeated with 0.5% Triton X‐100/1XPBS for 5 min, and rinsed repeatedly with 1XPBS. The samples were then incubated with 1% BSA/1XPBS for 1 h before incubation with primary antibodies overnight or 1 h. After washing with PBS, secondary antibody was added to the samples for 1 h. The secondary antibody was washed again three times with PBS, and the cell nuclei were then counterstained with DAPI (Merck). Epifluorescence microscopy was done in an inverted Olympus IX70, and confocal microscopy was done in a Zeiss Axioscope 2 Plus microscope equipped with a Bio‐Rad Radiance 2100 laser scanning system and Lasersharp 2000 imaging software and a Leica SP8 inverted focus microscope and analyzed with the leica application suite (las, Leica, Wetzlar, Germany) software. Autopsied animals were examined under a Leica M205FA fluorescent stereomicroscope, carrying a Leica DFC310FX camera and analyzed with leica application suite (las) software.

### 2.12 Immunohistochemistry in paraffin sections

Tissues isolated from experimental animals were fixed with 10% formalin (Merck) and kept overnight at 4 °C. They were then washed three times with sterile PBS and placed in 70% sterile ethanol (analytical grade). They were then placed in a sequential dehydration and paraffinization machine, and finally, the tissues were placed in paraffin to be cut into a microtome and stained with hematoxylin–eosin as described in a previous study [22]. If required, sections were stained with antibody CD31 (AbCAM, Cambridge, UK) and stained area was measured with imagej, National Institutes of Health and the Laboratory for Optical and Computational Instrumentation (LOCI, University of Wisconsin, Madison, WI, USA) as percentage of Microvessel density.

### 2.13 *In vivo* experiments on experimental animals

Tumor xenografting was done in 8–12‐week‐old female NOD.Cg‐Prkdcscid Il2rgtm1Wjl/SzJ (NSG) mice. In the case of MCF7 cells, β‐estradiol (8 μg·mL^−1^) was added to the water and was being replaced every 5 days until termination of the experiment. Mice were maintained in sawdust‐bedded cages and had a free access to food and water and a 12/12‐h light–dark cycle, at 22 °C and 50% humidity with 10 air changes/h. Tumor volume was estimated using the formula *V* = ½ (Length × Width^2^) [23]. To better monitor the engrafted tumor cells, control and KD PML cells were previously modified to express the red fluorescent mCherry protein. Equal cell numbers from either group were injected subcutaneously into NSG mice, and measurements from the growing primary tumors were taken every 4 days. When tumors reached 1200 mm^3^ in size, mice were sacrificed.

### 2.14 Approval of animal experiments

All procedures were conducted according to Greek national legislations and institutional policies following approval by the FORTH ethical committee. NSG mice were purchased from Jackson Laboratory (Bar Harbor, Maine, USA) and were maintained and bred at the Institute of Molecular Biology and Biotechnology (IMBB) animal facility following the institutional guidelines based on the Greek ethical committee of animal experimentation. Current procedures were approved by the General Directorate of Veterinary Services, Region of Crete (license number: 93304 and 106336).

### 2.15 Statistical analysis

The statistics were performed with microsoft excel software, (Microsoft Corporation, One Microsoft Way, Redmond, WA, USA) xlstat, (Addinsoft, Paris, France) and graphpad prism (Dotmatics, Boston, MA, USA). Values were presented as the mean ± SD from two or more experiments. For comparisons, the two‐tailed *t*‐test, Mann–Whitney, or *χ* ^2^ tests were performed as described in the results.

## 3 Results

### 3.1 Silencing of PML affects tumor cell morphology and physiology

**Fig. 1:**
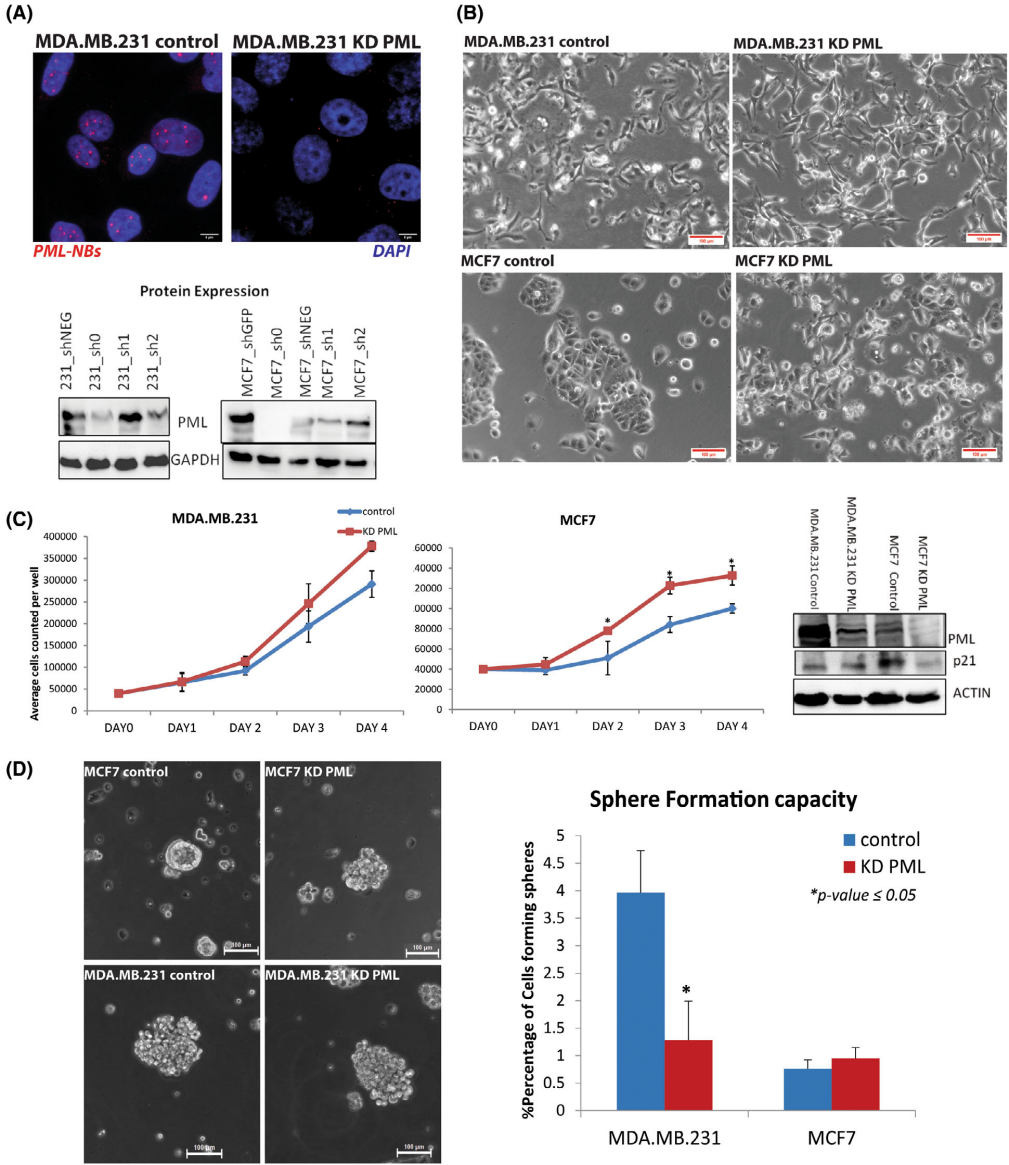
PML silencing alters breast cancer cell phenotype. (A) Upper: Confocal microscopy of PML Immunostaining (red nuclear speckles) in control and PML‐KD MDA‐MB‐231 cells counterstained by DAPI (blue; scale bar 8 μm). Shown are representative photographs of three experiments with similar results. Lower: Immunoblotting for PML protein expression of puromycin‐selected pools of MCF7 and MDA‐MB‐231 using a sh‐scrabbled control and three different shPML (sh0, sh1, and sh2) targeting sequences. Representative results of three experiments with similar results All further experiments were performed using the most efficient sh0 sequence. (B) Phase‐contrast microscopy of MDA‐MB‐231 KD PML & MCF7 KD PML compared with sh‐scrabbled controls (scale bar 100 μm). Shown are representative images of four independent observations. (C) Cell growth of control and PML‐KD, MDA‐MB‐231 (left), and MCF7 (middle) cells. Results show mean ± SD from one out of three independent triplicate experiments with similar results. *t*‐Test, *P‐value ≤ 0.05. Right: Immunoblot of p21 protein levels (CDKN1A) in control and PML‐KD cell lines. (D) Morphology of 3D tumor spheres of control or PML‐KD, MCF7, and MDA‐MB‐231 (left) and tumor sphere‐forming efficiency (right; scale bar 100 μm). Shown is mean ± SD of % sphere‐forming cells from one out of three quadruplicate experiments with similar results. *t*‐Test *P‐value ≤ 0.05.

To dissect the role of PML in breast cancer, we initially employed a doxycycline‐inducible shRNA approach that resulted in 50–55% reduction of the various PML isoforms. Thus, we switched to a constitutive knockdown approach by testing three different shRNAs (sh0, sh1, and sh2) for PML silencing in MDA‐MB‐231 and MCF7 cells. Knockdown (KD) efficiency was verified by qRT‐PCR (not shown), immunostaining, and western blotting using an antibody against a region common to all PML isoforms (Fig. 1A). Initially, we tested the tumor xenograft growth ability of PML control along with the MDA‐MB‐231 KD PML lines #0 and #2 that showed a significant reduction of PML expression. Figure S1 shows that sh0 and, to a lesser extent, sh2 caused a faster tumor growth relative to controls in line with their PML‐KD protein expression effect. Thus, we chose to use line KD sh0 for all further *in vivo* and *in vitro* studies shown here.

Microscopic examination of control and KD cells showed moderate changes of cell morphology in sparse cultures, toward a more spindle‐like shape of MDA‐MB‐231 (Fig. 1B). Similarly, MCF7 acquired a less tight epithelial cell‐to‐cell adhesion pattern. PML silencing slightly increased the proliferation of MDA‐MB‐231 and, to a larger extent, MCF7 in agreement with reduced expression of the cyclin kinase inhibitor p21 protein (Fig. 1C). We next tested the impact of PML ablation on cells grown in 3D as tumor spheres. Parental MCF7 and claudin‐low MDA‐MB‐231 formed compact‐round and grape‐like spheres, respectively [24]. The MCF7‐KD PML spheres assumed a more loose morphology that tends to resemble those of MDA‐MB‐231 cells (Fig. 1D). Most importantly, the sphere‐forming efficiency of MDA‐MB‐231–KD PML cells, but not that of MCF7 cells was reduced to about 30% of the control cells (Fig.1D).

**Fig. 2:**
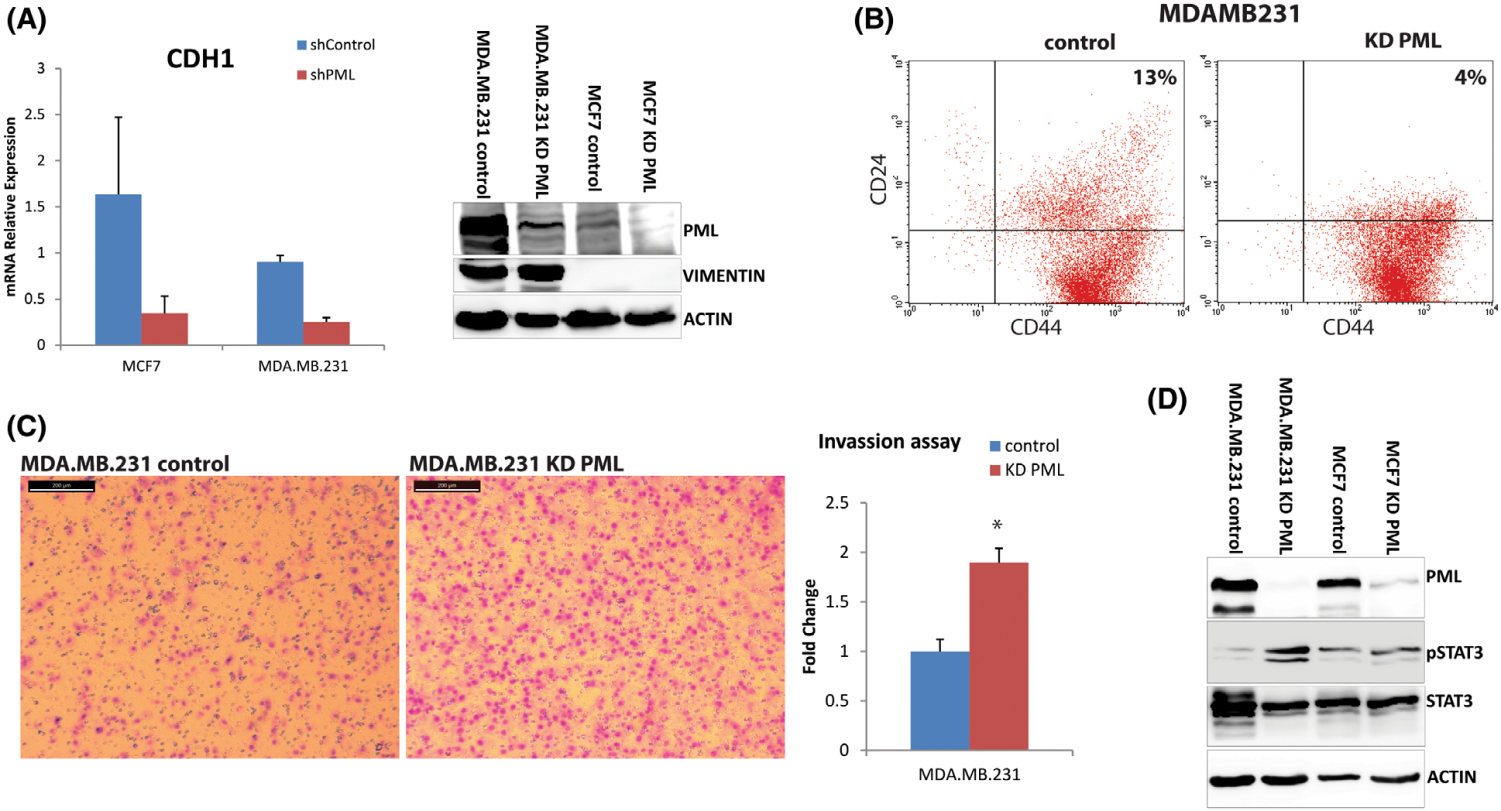
PML loss enhances mesenchymal properties in breast cancer cells. (A) qRT‐PCR of MDA‐MB‐231 and MCF7, control and PML‐KD cells of CDH1 expression (left). Results are mean ± SD (*n* = 3). Protein expression of a mesenchymal marker (Vimentin; right). Actin is loading control. (B) Shown is a representative flow cytometry for CD44 and CD24 surface expression in control and PML‐KD MDA‐MB‐231 (*n* = 2). (C) Microscopy of migrated, MDA‐MB‐231 control and PML‐KD cells (scale bar 200 μm). Fold change of % crossing cells ±SD (*n* = 3 triplicates). *t*‐Test *P‐value ≤ 0.05. (D) Representative Immunoblot for pSTAT3 protein (Tyr‐705) and total STAT3 (*n* = 2).

We have shown previously that PML is essential for maintaining the epithelial characteristics of naïve mouse embryonic stem cells [8]. The MDA‐MB‐231 cell line has mesenchymal characteristics, expressing high levels of VIMENTIN compared with MCF7 cells (Fig. 2A). Conversely, MCF7 express high levels of the epithelial marker CDH1 as compared to MDA‐MB‐231 cells. Following PML‐KD, we observed a decrease in CDH1 RNA in both MDA‐MB‐231 and MCF7 cells (Fig. 2A/left). At the protein level, the VIMENTIN expression was slightly increased in the former whereas the CDH1 was decreased in the latter cells (Fig. 2A/right & Fig. S2A). MDA‐MB‐231 cells express high levels of the mesenchymal marker CD44 and intermediate levels of the epithelial marker CD24, a phenotype early on linked to high tumor‐initiating (or cancer stem cell) ability of patient‐derived breast cancer xenografts [25]. Flow cytometry analysis for those surface markers showed that PML loss minimally affected CD24 or CD44 expression in MCF7 cells (Fig. S2B). However, PML‐KD in MDA‐MB‐231 led to a reduction of the CD24^+^ population (Fig. 2B). To further assess the epithelial/mesenchymal cell properties, we used an *in vitro* transwell assay and found that the absence of PML led to increased migration capacity of MDA‐MB‐231 (Fig. 2C) and to a lesser extent MCF7 cells (Fig. S2C). Moreover, the amount of phosphorylated STAT3 protein, known to correlate with TNBC aggressive behavior [26, 27], was increased (Fig. 2D).

**Fig. 3:**
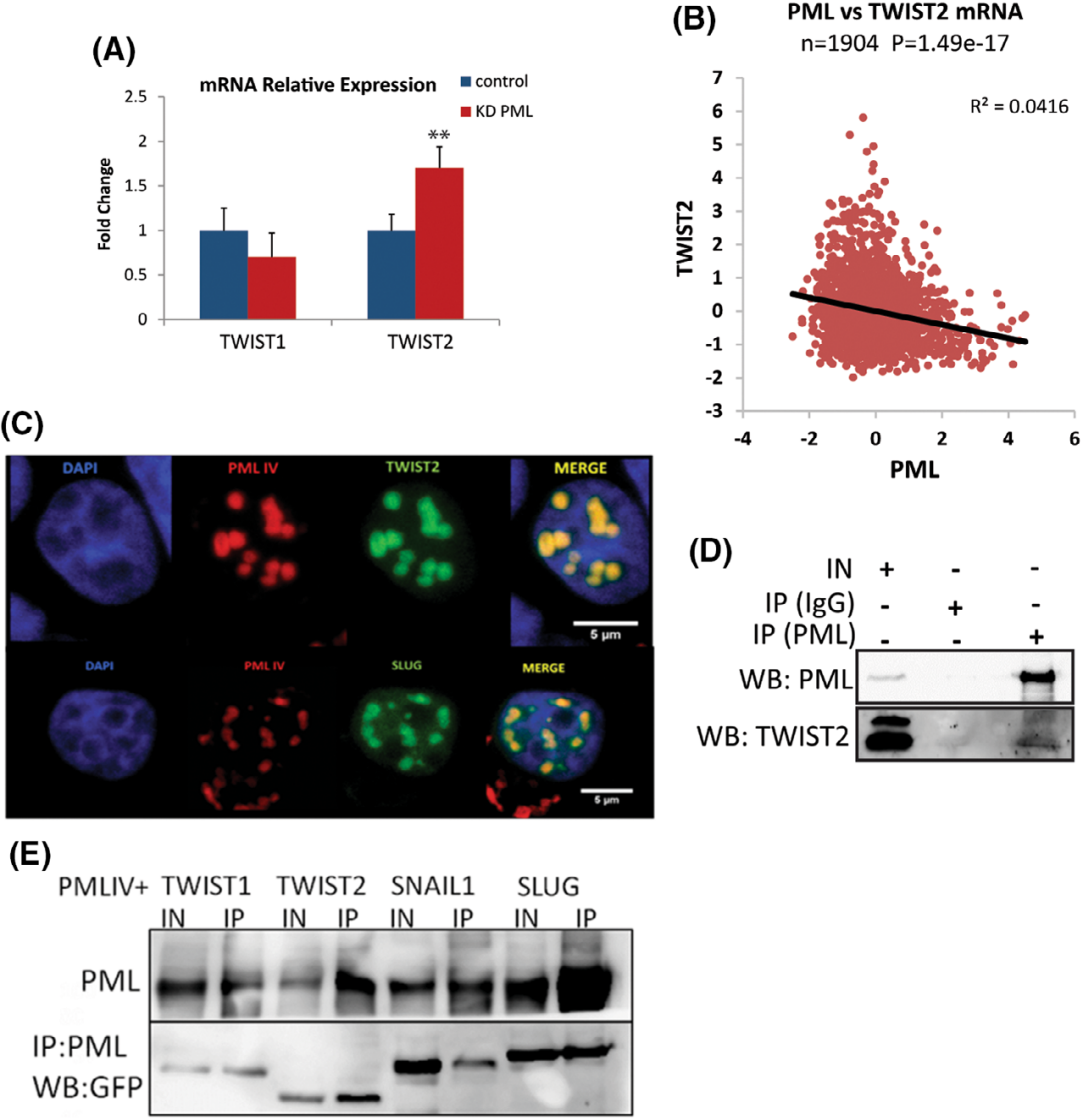
PML interacts with EMT factors and negatively correlates with TWIST2 expression. (A) qRT‐PCR of MDA‐MB‐231, control and PML‐KD cells for TWIST1 & TWIST2 expression. Results are mean ± SD (*n* = 3). *t*‐Test **P‐value ≤ 0.05. (B) Inverse correlation of PML–TWIST2 RNA expression levels (log_2_ microarray from the Metabric dataset). (C) Confocal images (representative of four experiments) of co‐localization between PML isoform IV, fused to mRED, and TWIST2 (upper panels) or SNAI2 (lower panels) fused to GFP (scale bar 5 μm). (D) Western blotting (WB) of co‐immunoprecipitation of endogenous PML and TWIST2 proteins in MDA‐MB 231 cells. Input (IN) or immunoprecipitated (IP) proteins were detected by an anti‐PML (IP : PML) or IgG (IP : IgG) used as negative control (*n* = 2). (E) Co‐immunoprecipitation of PML with TWIST1, TWIST2, SNAIL1, and SLUG (SNAI2) following co‐expression in HEK293T cells. Input (IN) or anti‐PML antibody immunoprecipitated (IP) proteins were detected by anti‐GFP (WB : GFP; *n* = 2).

Thus, PML loss seems to intensify the existing mesenchymal phenotype while reducing the epithelial phenotype of MDA‐MB‐231 cells, whereas in MCF7 cells, leads to a decrease in the CDH1 epithelial marker without inducing mesenchymal marker expression. Because epithelial–mesenchymal transition (EMT) transcription factors (EMT‐TFs) are mediators of the above phenotypic changes, we measured RNA levels of TWIST1 and TWIST2 key EMT factors and observed that PML loss caused a significant increase in TWIST2 RNA expression. In line with this, the TCGA breast cancer (Metabric) dataset [28] shows a strong inverse correlation of RNA expression between PML and TWIST2 (Fig. 3B).

Because PML protein isoforms have multiple interactor protein partners with diverse functional roles, we first examined whether TWIST protein co‐localizes with PML nuclear bodies using co‐expression of GFP or RFP fusion proteins and found strong co‐localization between PML IV and TWIST2 or SLUG (SNAI2; Fig. 3C). To examine the PML‐TWIST interaction in a more physiologic setting, we used co‐immunoprecipitation of MDA‐MB 231 cell extracts and found that anti‐PML, but not a control IgG, co‐precipitated TWIST2 protein (Fig. 3D). We extended these studies to TWIST1 and SNAIL1 EMT factors, expressed in HEK293T cells. PMLIV strongly co‐precipitated with all EMT factors but more intensely with TWIST2 and SNAI2 (Fig. 3E), whereas the PML III or PML I isoforms or an IgG control did not (Figs S3 right & S4 right). To define the region involved in the protein–protein interaction, we expressed TWIST2 truncations along with PMLIV followed by confocal microscopy and co‐immunoprecipitation. Confocal results showed localization with the central region of TWIST2 that includes the bHLH (Fig. S4 left). Indeed, the HLH region alone (aa64 to aa124) showed a strong co‐IP signal whereas the N‐ or C‐terminal parts had minimal or no interaction ability (Fig. S4 middle).

### 3.2 Changes in gene expression induced by PML ablation in breast cancer cells

**Fig. 4:**
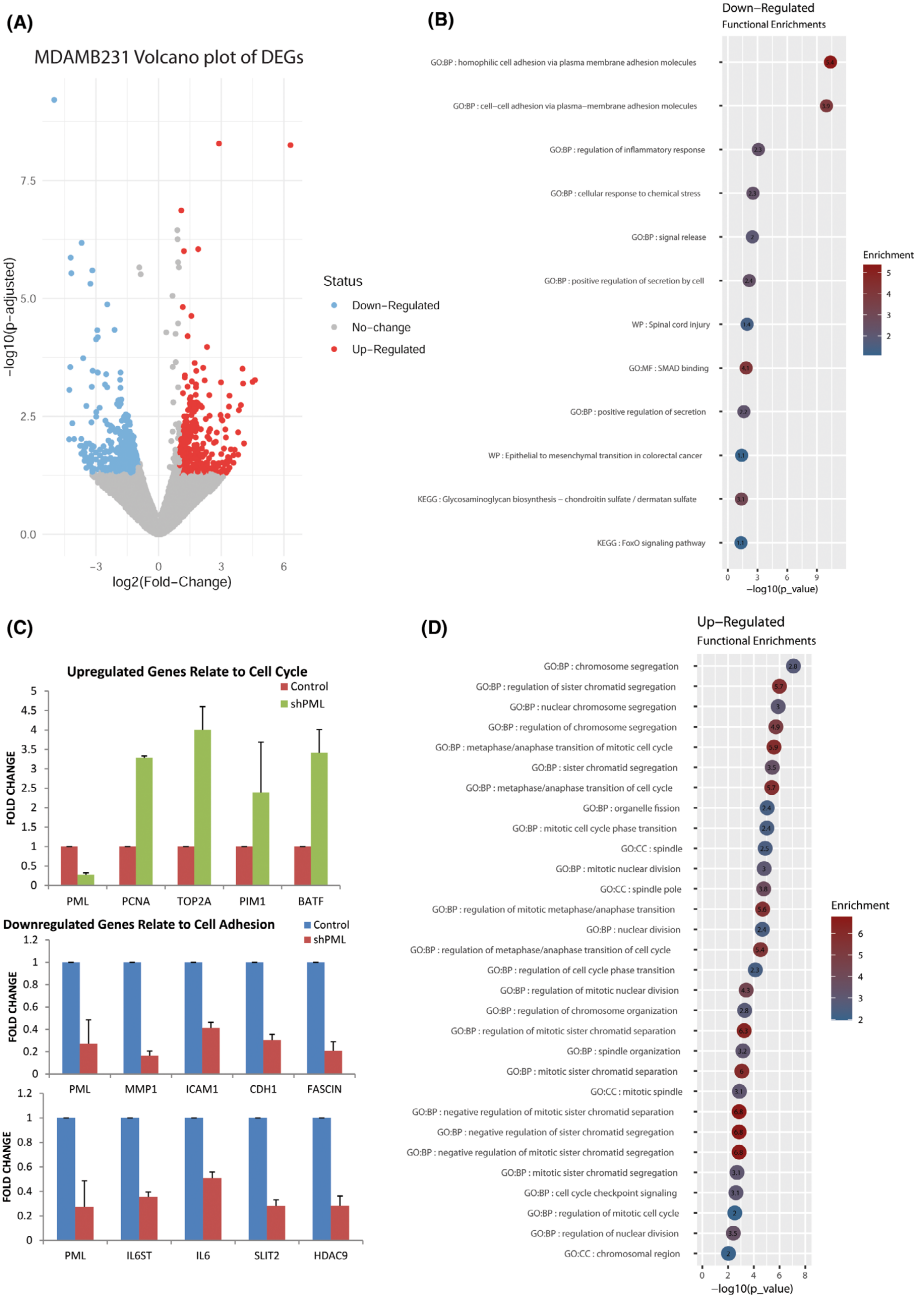
PML‐KD deregulates expression of genes related to cell cycle and cell adhesion. (A) Volcano plot of differentially expressed genes in MDA‐MB‐231 KD PML (*n* = 3), compared with control cells (*n* = 3) with *P*‐value ≤ 0.05, and log_2_ (FC) ≥ 1. (B) Top enriched (−log_10_ *P* values and fold enrichment) cellular processes of upregulated or downregulated (D) genes by PML loss in MDA‐MB‐231 cells. (C) Genes validated by qRT‐PCR. Results are mean ± SD (*n* = 3).

To correlate the above changes in cell behavior with the potential effects of PML loss in gene expression, we performed transcriptomic analyses of control and KD PML cell lines. Specifically, silencing of PML deregulated about 600 genes at the 1.5‐fold cutoff in MDA‐MB‐231 cells. Upregulated genes were enriched in cell cycle and cell division, whereas downregulated genes showed enrichment in cell adhesion functions, EMT and microRNAs that target tumor suppressors (Fig. 4). A similar analysis of control and KD PML MCF7 cells identified 736 down‐ and 256 upregulated genes. Upregulated genes were enriched in differentiation, morphogenesis, locomotion, and cell adhesion terms that involve NFKB1, RELA, and HIF1A factors, while downregulated genes involve ECM organization and cytokine signaling. Validation of deregulated expression for a number of those genes was done by qRT‐PCR analysis (Fig. S5).

Comparisons of the two cell types showed little overlap in deregulated genes indicating a cellular context‐specific response to PML loss. Common GO terms were related to stress response and developmental processes.

### 3.3 PML affects growth and hypoxic response of tumors derived from MDA‐MB‐231 xenografted cells

**Fig. 5:**
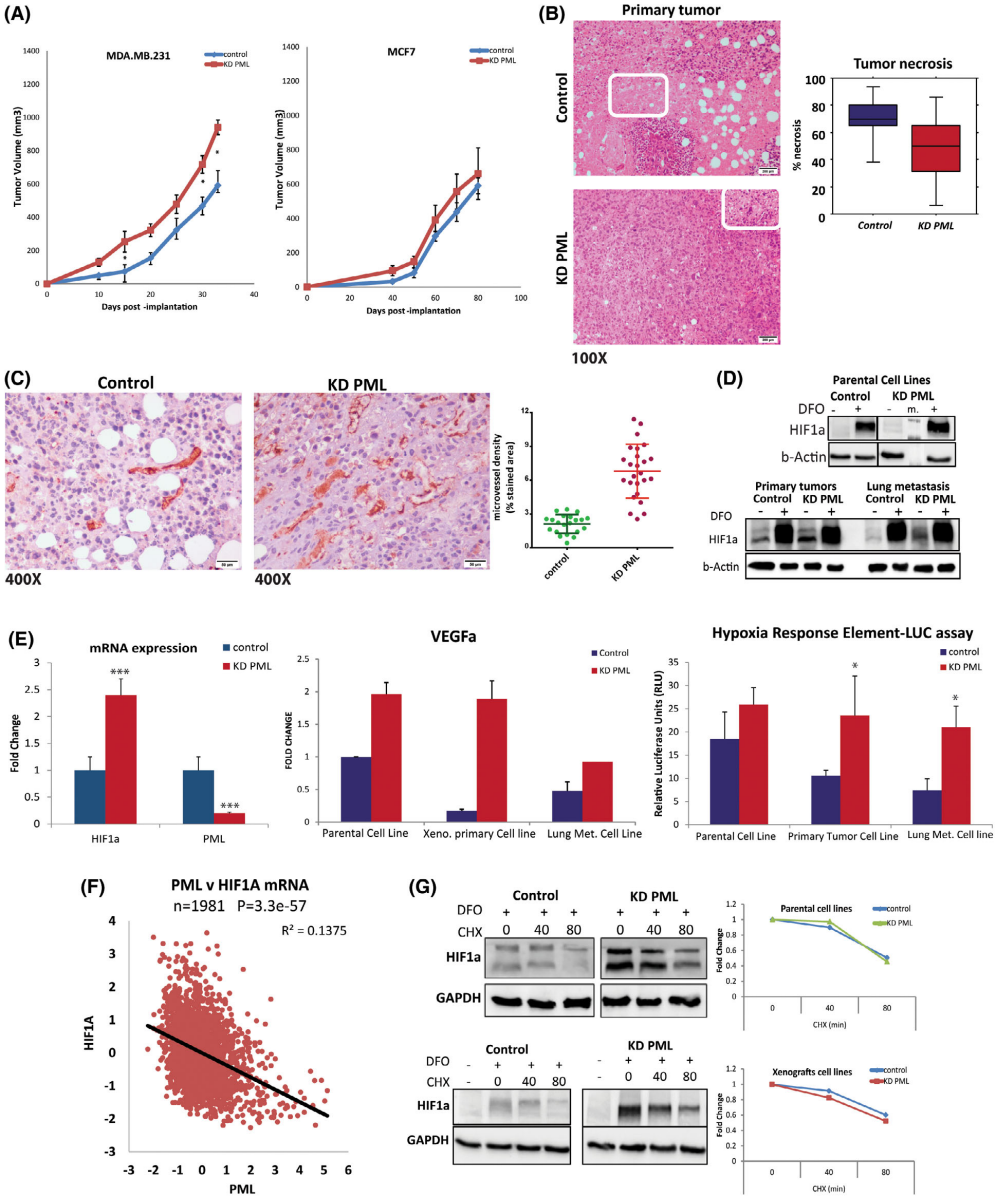
PML loss increased *in vivo* primary tumor growth and vascularization via HIF1a. (A) Tumor growth of MDA‐MB‐231 and MCF7, control (blue, *n* = 7) & KD PML (red, *n* = 7). Results show mean volume ± SD from one of two independent experiments with similar outcomes. *t*‐Test *P‐value ≤ 0.05. (B) Hematoxylin–eosin at 100× magnification of primary MDA‐MB‐231 control & KD PML tumors (left). Rectangles indicate necrosis (scale bars 200 μm). Quantitation of primary tumor necrosis is presented by box plots of % necrotic area of primary tumors (*n* = 7/group, Mann–Whitney *U*, *P‐value ≤ 0.05; right). Error bars show minimum/maximum values (right). (C) CD31 staining at 400× magnification of primary MDA‐MB‐231 control & KD PML tumors (left; scale bar 50 μm). Quantitation (mean ± SD) of primary tumor staining with anti‐CD31, indicative of angiogenesis (*n* = 21 areas for control group and 25 areas for KD PML group/2–5 representative areas per tumor/5 tumors; right), *t*‐test *P‐value ≤ 0.001). (D) Immunoblot for HIF1α protein in parental and xenograft‐derived lines from primary or lung metastatic MDA‐MB‐231 cells with or without DFO (Desferrioxamine 100 μm) treatment for 12 h. (E) Comparative expression of *HIF1Α* gene in parental cell lines (left) and *VEGFΑ*, a gene target of HIF1α, in parental or xenograft‐derived cell lines. Results are mean ± SD (*n* = 3; middle). HRE‐LUC assay of control or PML‐KD parental cell lines, and primary tumor site or lung metastasis‐derived cell lines. Results are mean ± SD (*n* = 2 triplicates; right). *t*‐Test *P‐value ≤ 0.05, ***P‐value ≤ 0.01 (F) Inverse correlation of *PML–HIF1Α* RNA expression levels (log_2_ microarray from Metabric dataset). (G) Representative (*n* = 2) western blot of HIF1α in parental and xenograft‐derived lines from primary MDA‐MB‐231 cells treated with DFO for 4.5 h followed by treatment with cycloheximide (CHX). Graphs express HIF1α levels upon normalization for β‐ACTIN as fold over cells treated with CHX after DFO.

To study and correlate the above results with the *in vivo* tumor growth of PML‐silenced cells, we performed engraftment in NSG mice. PML loss led to an increase in the tumor growth rate in MDA‐MB‐231 cells, but not MCF7 cells (Fig. 5A). Histology of primary MDA‐MB‐231 KD PML tumor sites showed reduced necrosis relative to control group (Fig. 5B). To test whether reduced necrosis might be due to enhanced vascularization, we used anti‐CD31 antibody staining in sections of primary site tumors. KD PML tumors showed significantly higher microvessel density relative to the control group (Fig. 5C). To further study these cells after their *in vivo* passage, we readapted tumor tissue back into cell culture and developed more than 16 lines from primary or metastatic sites. We examined the basal and desferrioxamine (DFO)‐induced HIF1A protein of parental and xeno‐derived lines and found that KD PML had a twofold higher basal HI1A expression that was further intensified following *in vivo* growth (Fig. 5D). Luciferase assays by transient transfection of the 5xHRE luc hypoxia response corroborated these results (Fig. 5E right). PML loss resulted in a similar increase in HIF1A RNA and VEGF‐α, a target of HIF1a and a main angiogenic factor, in both the parental and xeno‐derived KD PML lines, (Fig. 5E left). In agreement, inspection of clinical breast cancer data from TCGA–Metabric showed a striking inverse correlation of PML and HIF1a (Fig. 5F). Taken together, these results suggest that PML loss facilitates HIF1A RNA expression to promote the acquisition of a hypoxic phenotype that is further enhanced in a hypoxic primary site relative to the lung metastatic growths. Because HIF1a is well known to be regulated at the protein stability level, we next studied protein turnover in control and KD PML cells first induced with DFO and next with cycloheximide (CHX). Similarly to basal HIF1A protein levels, DFO‐mediated inhibition of degradation resulted in a proportional twofold increase in both parental and xeno‐derived, either control or KD PML lines. A follow‐up inhibition of protein synthesis by cycloheximide showed that PML loss did not affect the protein degradation rate (about 80 min half‐life). Overall, the above indicates that PML loss promotes hypoxia response by mainly affecting both the RNA and protein expression of *HIF1A* but not turnover (Fig. 5G).

### 3.4 The ablation of PML regulates the metastatic potential and organ preference of MDA‐MB‐231 cells

**Fig. 6:**
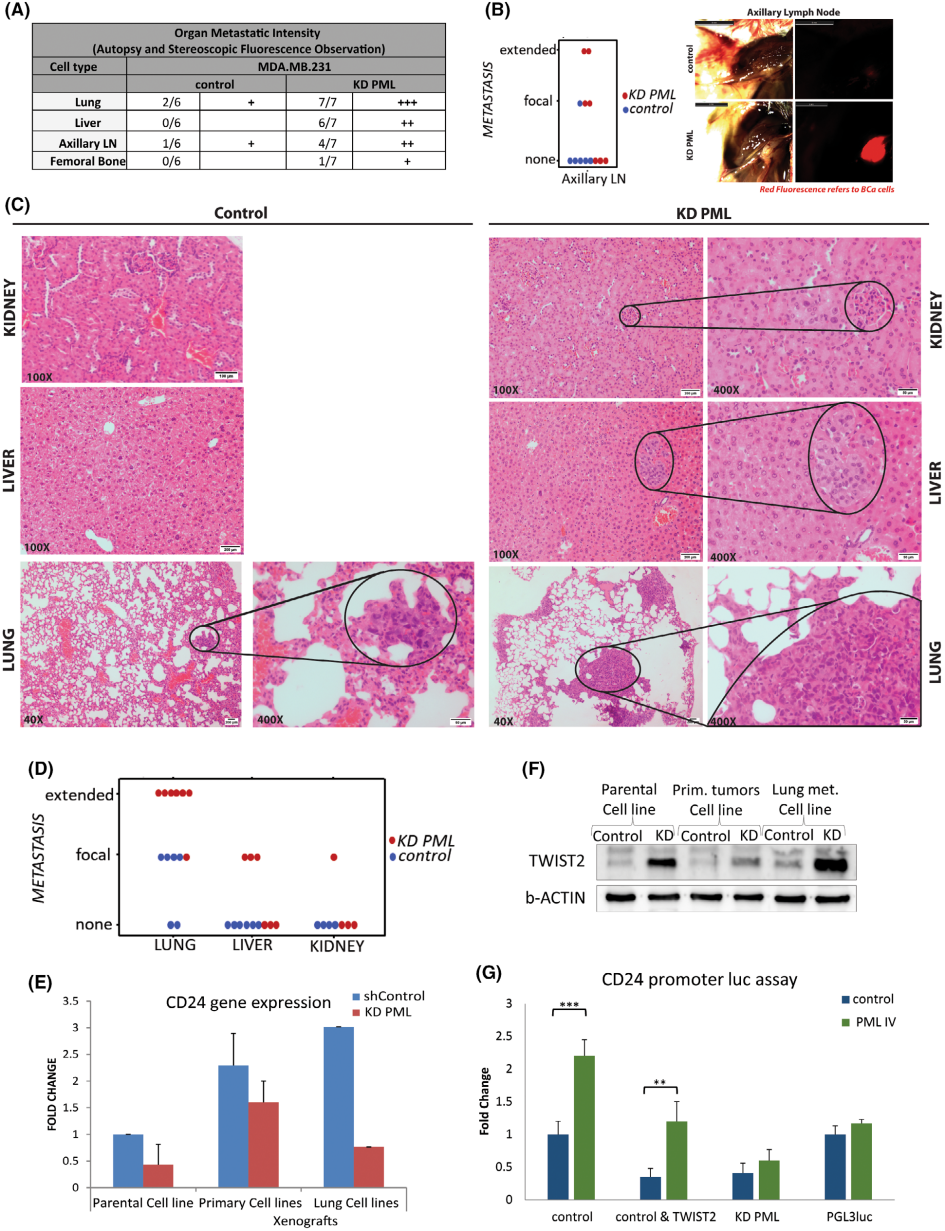
PML loss exacerbated the metastatic phenotype of MDA‐MB‐231 cells. (A) Summary table of metastases in mice with MDA‐MB‐231 control & MDA‐MB‐231 KD PML cells and grading by stereoscopic observation as +: positive, ++: focal, and +++: extensive. (B) imagej quantification of axillary gland metastasis in control (*n* = 6) and PML‐KD (*n* = 7) by fluorescence stereomicroscopy (*χ* ^2^ test *P*‐value ≤ 0.05). Representative axillary gland metastasis imaged by visible or fluorescence stereomicroscopy (right; scale bar 5 mm). (C) Representative hematoxylin–eosin staining of kidney and liver (100× magnification, scale bar 100 and 200 μm) and lung histologies of control and PML‐KD‐engrafted mice (40× and 400× magnification, scale bar 200 and 50 μm). Encircled areas indicate metastatic foci. Sections are representative of five each of control and PML‐KD xenografts. (D) Quantitation and *χ* ^2^ test of metastatic load in control (*n* = 6) and PML‐KD (*n* = 7) engrafted mice for lung (*P* = 0.002), liver (*P* = 0.026), and kidney (*P* = 0.28).(E) CD24 mRNA expression in parental and xenograft‐derived cell lines. Results are mean ± SD of two triplicates. (F) TWIST2 protein expression in parental and xenograft‐derived cell lines. Shown are results of one experiment (*n* = 2). (G) Luc CD24 promoter activity in control or TWIST2 cotransfected, untreated, or doxycycline(dox)–induced PML IV expressing cells. Results show mean ± SD from two triplicates. Shown significant are *t*‐test values only: ***P‐value ≤ 0.005, **P‐value ≤ 0.05.

**Fig. 7:**
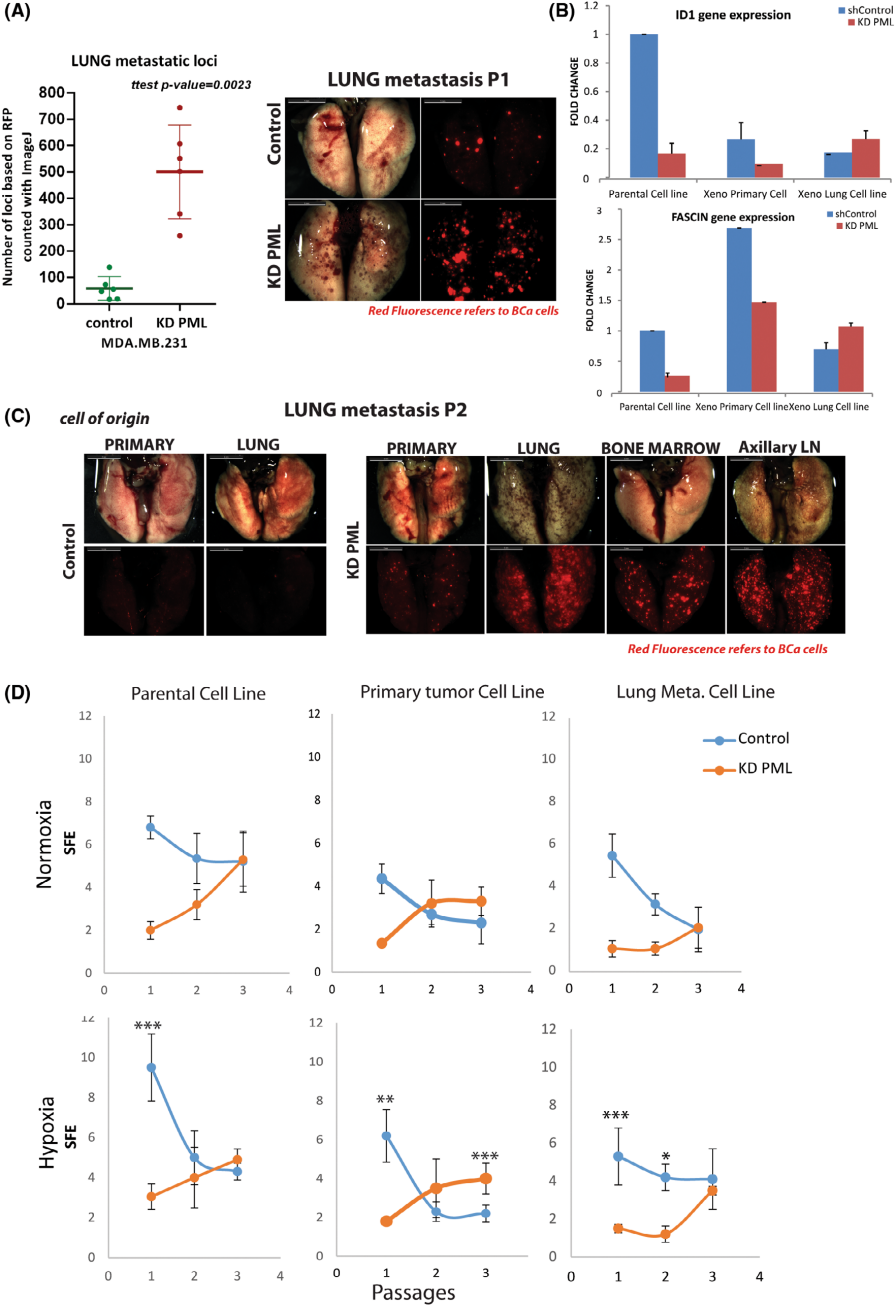
PML loss promotes lung metastasis and sustains long‐term TSF ability. (A) Assessment of lung metastatic load of control and PML‐KD‐engrafted mice by comparing (*t*‐test, *P*‐value ≤ 0.05) total foci counts (left) and representative stereomicroscopy image (middle; scale bar 5 mm). (B) Comparative expression of lung ‘metastasis signature’ [14] (LMS) genes *ID1* and *FSCN1* in control and KD PML, parental and xenograft derived cell lines. Results are mean ± SD from a triplicate experiment. (C) Representative (from *n* = 3 each of control and PML‐KD engrafted mice) stereomicroscopy from second passage (P2) lung metastases following reinjection of first passage‐derived cells (scale bar 5 mm). (D) Percentage (%) of cells forming spheres (SFE), when cultured under normoxic or hypoxic conditions from parental and xenograft‐derived cell lines through sequential passaging P1, P2, and P3. Results show mean SFE ± SD from one out of three quadruplicates. *t*‐Test.

Gross observation and stereomicroscopy of autopsied tumor‐bearing mice showed that all (7/7) the KD PML expressing, MDA‐MB‐231 tumors, had a more intense metastatic load relative to controls in all the organs examined. More specifically, all KD PML mice had many more lung metastatic foci, 6/7 were positive for liver metastases, 4/7 showed metastases in the axillary glands, and one (1/7) showed metastasis to the bone marrow (Fig. 6A) compared with tumors from control mice. These were further confirmed and quantified by stereomicroscopy and histology of axillary lymph node, liver, kidney, and lung samples (Fig. 6B–D). Of note, lung was the most extensively affected site observed (Fig. 6C,D) with an estimated 10‐fold higher metastatic load in the KD PML (see also Fig. 7A). MCF7 control or KD PML tumors showed no significantly different growth rate (Fig. 5A), and no metastasis was found in either group (Fig. S6). All PML control and KD lines derived from tumor tissue retained differential expression of PML RNA, and protein, similar to their parental cell lines (Fig. S7A,B). In addition, they showed higher expression levels *of* recurrence‐metastasis‐related markers, such as EpCAM [29] and CD49f [30] (Fig. S7C) as well as reduced epithelial CD24 RNA levels (Fig. 6E) and increased TWIST2 protein expression (Fig. 6F).

Because CD24 is a direct target of TWIST repression, we used a CD24 promoter driving Luciferase to functionally study the TWIST–PML interaction. Using transient transfection assays in MDAMD231 cells, we showed that transient expression of PML IV increased, whereas TWIST2 decreased activity. In addition, PML IV rescued TWIST2‐mediated repression (Fig. 6G). In a PML‐KD setting, reintroduction of PML partly rescued promoter activity (Fig. 6G) as anticipated because of the dampening effect of the shPML carried by the cells. Overall, PML opposes TWIST2 repressive activity on its target promoter in line with lower cell RNA (Fig. 6E) and surface protein expression (Fig. 2B).

Previous studies have shown that hypoxia can induce EMT [31]. We wanted to investigate whether PML acts on EMT factor activity via the hypoxia–HIF pathway. To this end, we knocked down HIF using an shHIF in control or KD PML MDA‐MB 231 cells. As expected, expression of both HIF1a and its major target VEGFa was compromised in control of KD PML cells (Fig. S8A). Moreover, doxycycline(dox)‐induced PML IV expression drastically reduced both HIF1a and VEGFa expression further supporting a negative role of PML in hypoxia signaling (Fig. S8B). Next, we tested whether HIF KD affected expression of known TWIST2 target genes, namely CD24 and CDH1 that are upregulated by dox‐inducible PML IV expression (Fig. S8C). Results show that HIF KD did not significantly alter their expression (Fig. S8D), suggesting that PML regulates EMT in an HIF‐independent way. Combined, these results suggest that KD PML cells are enriched in mesenchymal properties consistent with a pro‐metastatic phenotype because PML antagonizes EMT factors by acting at both their expression and protein activity levels.

The increased lung metastatic ability of MDA‐MB‐231 KD PML‐derived tumor lines (Fig. 7A) was maintained upon secondary xenografting (Fig. 7C). By qRT‐PCR analysis of characteristic lung metastasis signature genes [14], namely ID1 and FSCN1, using the original and tumor derived cell lines, we found that derived cells had higher expression than their parental cell lines (Fig. 7B). Because results of tumor sphere formation (TSF) ability seemed not to be in line with *in vivo* tumor growth, we chose to examine in more detail the TSF ability of parental and tumor derived MDA‐MB‐231 cell lines under normoxic or hypoxic conditions. Results from these experiments show that passage one (P1) KD PML from either parental or tumor‐derived lines had a lower TSF ability (20–30%) than controls, under either normoxic or hypoxic (3% oxygen) conditions, although in the latter a general increase in TSF was found. However, during subsequent passages the TSF ability of KD PML cells increased whereas that of the control groups decreased. At P3, parental, primary tumor and lung‐derived KD PML cells exceeded their controls in pairwise comparisons (Fig. 7D).

## 4 Discussion

To investigate the role of PML in breast cancer, we produced knockdown lines of the claudin‐low MDA‐MB‐231 and the luminal‐A MCF7 and studied their physiology and transcriptomic profiles.

Our previous studies have shown that induced overexpression of PMLIV in MDA‐MB‐231 cells strongly inhibited cell proliferation and tumor sphere formation (TSF) ability [11]. While PML silencing of all PML isoforms in those cell types caused a slight increase in the cell growth rate in monolayer culture, their tumor sphere ability was compromised (25–30% of the control line) implying a role for PML in their maintenance of stem‐cell‐like properties. In addition, PML loss changed the MCF7 tumor spheres from their known smooth spherical morphology [24] to a more grape one, reflecting a change in the expression of cell–cell interacting proteins. The ablation of PML caused a shift in both cell lines toward a more mesenchymal appearance that has been previously linked to increased cell invasiveness [32, 33] and tumor recurrence or metastasis [34, 35]. In accordance with this, PML‐deficient cells showed increased migration capacities. The increase in migration activity was more pronounced in MDA‐MB 231 cells and correlated with higher phosphorylated STAT3 [36].

To correlate these changes with RNA expression profiles, we report here for the first time a transcriptomic analysis upon silencing of PML. The transcriptomic analysis reflected the differences in the physiology of these cell types. The ablation of PML in MDA‐MB‐231 cells caused the repression of genes related to cell adhesion and ECM. On the contrary, genes related to cell cycle, mitosis, and cell division were upregulated. A similar analysis in MCF7 cells identified developmental–differentiation‐related genes to be upregulated and cytokine and extracellular matrix‐related genes to be repressed upon PML‐KD. Interestingly, earlier studies of transient PML‐KD in HUVEC cells identified some common GO‐enriched classes such as cell cycle and locomotion [37].

Together the phenotype and deregulated gene expression profile suggest that PML‐deficient MDA‐MB‐231 cells are expected to be more aggressive than their parental cells. This contrasts with the reduction in TSF that is thought to measure tumor‐initiating cells (TICs) or cancer stem cell activity. To clarify this, we xenografted control and KD PML cells in NSG mice and monitored their growth in the primary site and development of metastasis. PML‐KD did not affect significantly the local tumor growth or the non/low metastatic properties of MCF7 cells. However, MDA‐MB‐231 KD PML cells developed tumors faster when compared to the controls. This observation contradicts the *in vitro* TSF assay, suggesting that the growth defect in PML loss can be reversed during *in vivo* tumor growth. Indeed, upon sequential tumor sphere passaging, KD PML cells showed a gradual increase in their TSF ability concomitant with a decrease in the control TSF. These results show that KD PML cells retain higher long‐term TSF ability especially when taken from primary tumor sites and/or hypoxic conditions in agreement with their higher *in vivo* tumor growth rate and metastasis. Taken together, these results suggest that KD PML cells acquire higher TIC properties *in vivo* and show a more aggressive phenotype. Tissue hypoxia seems to further intensify this process. These results partly disagree, with the observations of Ponente et al. [38], Martin‐Martin et al. [39] and Arreal et al. [40] that describe a pro‐oncogenic role of PML in breast cancer. Although presently undetermined, these differences may be caused by distinct MDA‐MB‐231 subline differences or the effect of long‐term and strong PML silencing in our lines.

Epithelial–mesenchymal transition factors have important roles in normal development and are often found to be deregulated in cancer [41] in response to upstream activators [42]. The role of interaction of PML with EMT factors seems to be complex. Cytoplasmic PML promotes EMT via potentiation of TGF‐β signaling [43, 44]. We described here for the first time that both bHLH (TWIST) and Zn‐finger (SNAIL) type EMT factors bind and co‐localize to PML IV and to a lesser extent to PML I‐ and III‐driven nuclear bodies. Interaction with TWIST2 requires the presence of the bHLH domain and may result in altered dimerization or DNA binding in a way similar to the inhibitory action of IDs on EMT factors. Reversion of TWIST2‐mediated repression of the CD24 promoter by PML further lends support to an inhibitory role of the PML–TWIST binding. In addition, PML‐KD leads to increased TWIST2 RNA and protein expression in parental tumors and the lung metastases in line with a strong inverse correlation of PML and TWIST2 expression in a large breast dataset (TCGA–Metabric).

Earlier reports proposed that the HIF–hypoxia signaling transcriptionally activates EMT factors [31].To address the possibility that PML‐KD affects EMT via HIF activation, we produced double HIF KD lines on MDA‐MB‐231 of either control or PML‐KD background and examined the expression of HIF and EMT signaling. Our results show that HIF KD reduced basal as well as hypoxia‐induced (not shown) HIF RNA levels (in line with a previously reported auto‐regulatory loop [45] accompanied by reduced VEGF levels as expected. However, repression of TWIST targets CD24 and CDH1 was not affected, thus supporting a HIF‐independent role of PML in controlling EMT signaling in those genes. Future genome‐wide studies will determine whether this is a global or gene‐selective function of PML. For instance, we compared the deregulated gene lists of our KD PML cells with those of TWIST1‐overexpressing MCF7 cells [46] and observed a significant number of gene overlaps in both the up and down categories, enriched in distinct processes, suggesting complex mechanisms of PML‐EMT interactions. Interestingly, PML‐deficient MCF7 and MDA‐MB231 did not share any of these TWIST intersecting genes, indicating a cell type‐specific response of distinct TWIST‐regulated genes in different cell types.

An important observation of this study was the increased metastatic properties of PML‐deficient MDA‐MB‐231 cells to various sites and a stronger preference for the development of lung metastasis. Earlier studies have identified a group of genes in MDA‐MB‐231 cells expressed at higher levels in cell lines derived from lung metastases (lung metastasis signature, LMS) relative to their parental cell line [14]. The differentially expressed KD PML genes and the LMS list contain 11 common genes (shown in Table S3). Interestingly, FSCN1 and ID1 showed a change in their expression pattern resembling that of LMS. The ID gene family has important roles in development and their deregulated expression is connected to poor prognosis. Increased expression of ID1 has been associated with both primary tumor formation and lung colonization [47] presumably via mesenchymal–epithelial transition essential for the latter process [48]. Earlier studies demonstrated that MDA‐MB‐231 are genetically heterogeneous and *in vitro* selection for more aggressive populations with altered genome structure has been reported [49]. Similarly, sequential *in vivo* growth of MDA‐MB‐231 selects for highly metastatic sublines enriched in preexisting or newly arising genetic variants such as BRAF and KRAS [50] as well as ALPK2 and RYR1 [51] that correlate with high organ‐specific metastatic ability. Although not directly demonstrated, it is likely that PML deficiency accelerates this *in vivo* selection process. In the present studies, PML affected both the primary and metastatic tumor growth. Searching for metastatic suppressor genes (MSG), we found that the ablation of PML resulted in the up‐ or downregulation of a small number of MSGs [52] but their functional significance remains to be determined.

Tumors produced by MDA‐MB‐231 KD PML cells showed lower necrotic content suggesting higher vascular supply. In agreement with this, CD31 immunocytochemistry showed a significant increase in microvessel density in KD PML primary tumors. In agreement with this, parental or lines generated by culturing KD PML tumors expressed higher levels of HIF1a protein and its target, VEGFa that may account for a higher tumor angiogenic activity. We show here that PML loss enhanced HIF1a and consequently VEGFa RNA expression without affecting the protein turnover unlike its role in promoting HIF1a protein synthesis in a mouse KO model [53].

## 5 Conclusions

Cell and transcriptomic analysis show that PML impedes tumorigenicity by regulating cell proliferation, the mesenchymal‐invasive and angiogenic properties, and the metastatic ability of MDA‐MB‐231 cells. Mechanistically, PML is shown to inhibit the mesenchymal phenotype by negatively regulating both the expression and the gene‐repressive activity of TWIST2. In addition, PML loss promotes hypoxia–angiogenic switch that favors local and metastatic growth as well as the long‐term maintenance of tumor‐initiating cells. Finally, our data suggest that PML loss may accelerate *in vivo* evolution of genetically heterogeneous population to acquire a more aggressive‐metastatic phenotype. Hence, PML expression may serve as a cancer biomarker.

## Conflict of interest

The authors declare no conflict of interest.

## Author contributions

APV designed and performed experiments, data analysis, discussion, and writing; SS, NS, and TM contributed to the experimental work and discussion; ED performed immunohistochemistry in paraffin sections and histological analysis; CN performed bioinformatic analysis; AK contributed to discussion and writing; JP supervised the study and contributed to writing.

## Supporting information


**Fig. S1.** (related to Fig. 5): Increased *in vivo* tumor growth of MDA‐MB‐231 PML‐KD lines by two different shRNAs relative to controls.
**Fig. S2.** (related to Fig. 2): PML loss enhances mesenchymal properties in MCF7 breast cancer cells.
**Fig. S3.** (related to Fig. 3): PML I & III isoforms examined for interaction with TWIST2.
**Fig. S4.** (related to Fig. 3): PML IV interacts specifically with the bHLH domain of TWIST2.
**Fig. S5.** (related to Fig. 4): Bioinformatics analysis and validation of MCF7.
**Fig. S6.** (related to Fig. 6): MCF7 control and KD PML cells showed no metastatic lesions.
**Fig. S7.** (related to Fig. 6): Xenograft‐derived cell lines maintained PML silencing and showed increased expression of highly aggressive metastasis‐prone tumor‐related markers.
**Fig. S8.** (related to Fig. 6): PML independently suppresses HIF1a and TWIST2 signaling.

**Table S1.** Primers used for qRT‐PCR (related to Section 2.4).
**Table S2.** Antibodies used in western blots and Co‐IPs (related to Sections 2.7 and 2.8).

**Table S3.** (related to Fig. 7): List of 15 common genes between lung metastasis signature gene set [14] and MDA‐MB‐231 DEGs.

## Data Availability

RNA‐seq datasets have been deposited in SRA database under accession number PRJNA826854.
