# Supplementary material for: Promyelocytic leukemia protein regulates angiogenesis and epithelial–mesenchymal transition to limit metastasis in MDA‐MB‐231 breast cancer cells
Source: Mol Oncol. 2023 Sep 4;17(10):2090–108. doi: 10.1002/1878-0261.13501 (PMC10552902; doi:10.1002/1878-0261.13501)

<u>Figure S1 (related to figure 5):</u> Increased *in vivo* tumor growth of MDA-MB-231 PML-KD lines by two different shRNAs relative to controls. Results show mean tumor volume ±SD at the indicated post-grafting time points. sh0 (red, n=7), sh2 (pink, n=6) and control shNEG (blue, n=6). ****t-test p-value≤ 0.01,* $^{\#}$ *t-test p-value≤ 0.05*

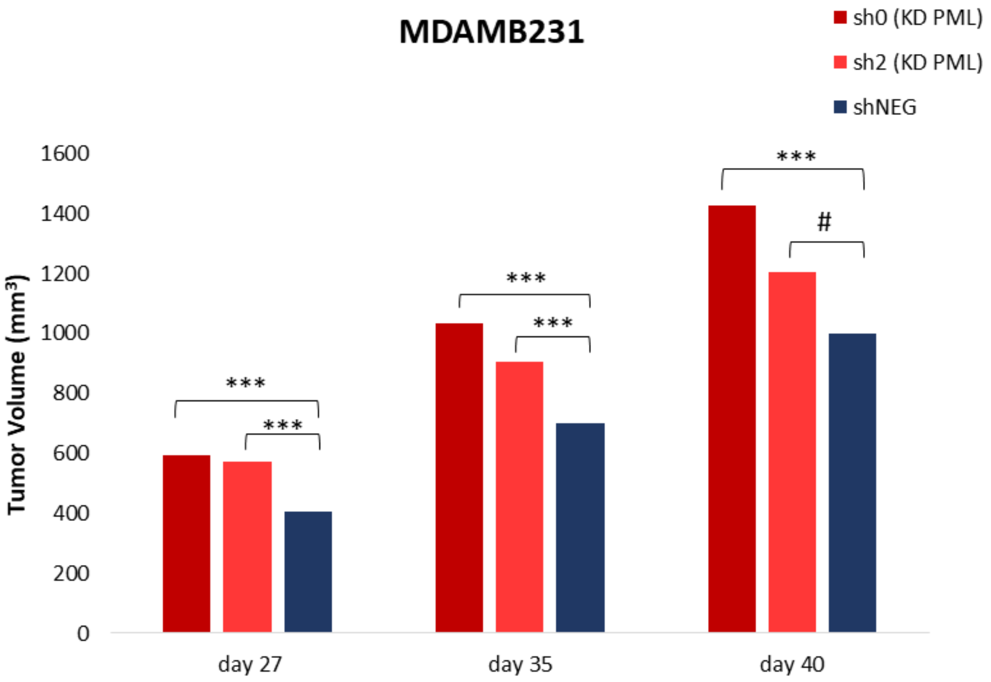

Figure S2 (related to figure 2): PML loss enhances mesenchymal properties in MCF7 breast cancer cells. (A) Protein expression of an epithelial marker (CDH1) and estrogen receptor a (ERa). GAPDH is loading control. (B) One out of two similar flow cytometry experiments for CD44 and CD24 surface expression in control and PML KD MCF7 cells. (C) Microscopy of migrated, MCF7 control or PML KD cells (Left panels). Scale bar is 200µm. Fold increase of (%) crossing PML –KD cells. Mean ±SD of one out of two triplicate experiments with similar results (Right). *t-test *p-value≤0.05.*

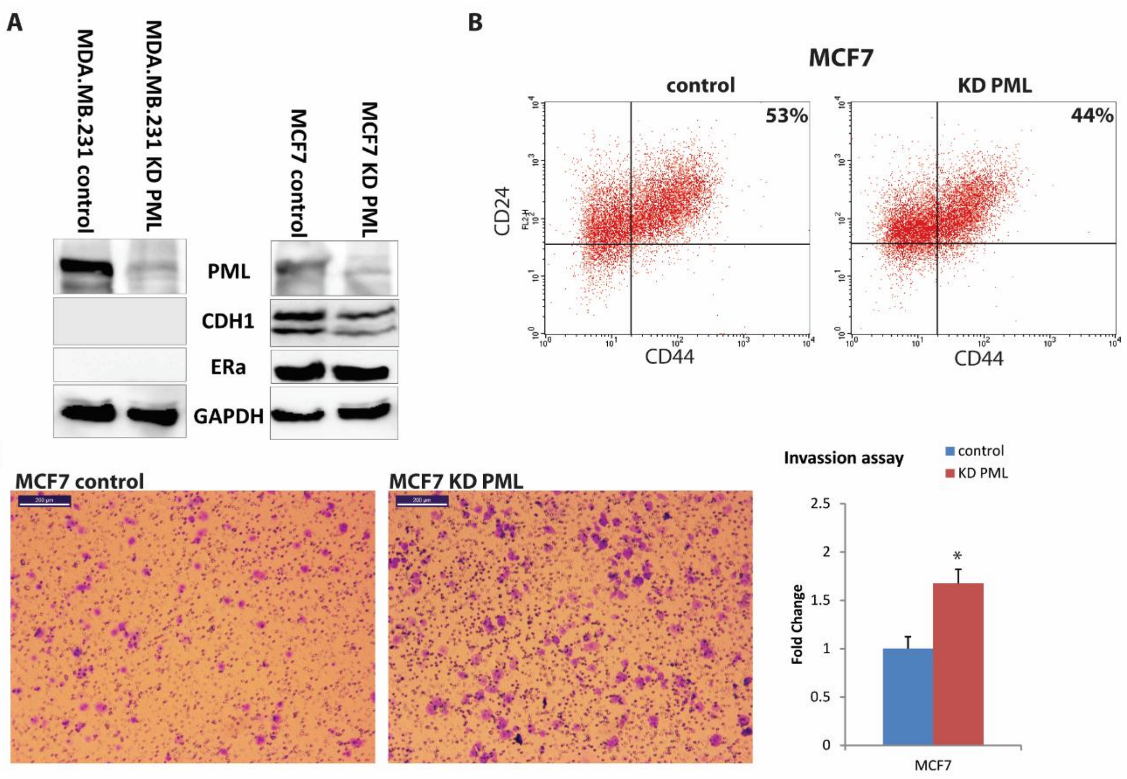

<u>Figure S3 (related to figure 3):</u> PML I & III isoforms examined for possible interaction with TWIST2. Left: Co-localization of PMLI (lower panel) & III (upper panel) and TWIST2 by a confocal microscopy PMLI & III (red), TWIST2 (green). Right: Immunoprecipitation by anti-PML antibody of PML I or III isoforms along with TWIST2-GFP protein in HEK293T cells, followed by Western blot analysis by anti –GFP. Shown one out of at least two independent experiments with similar results. Scale bar 5µm.

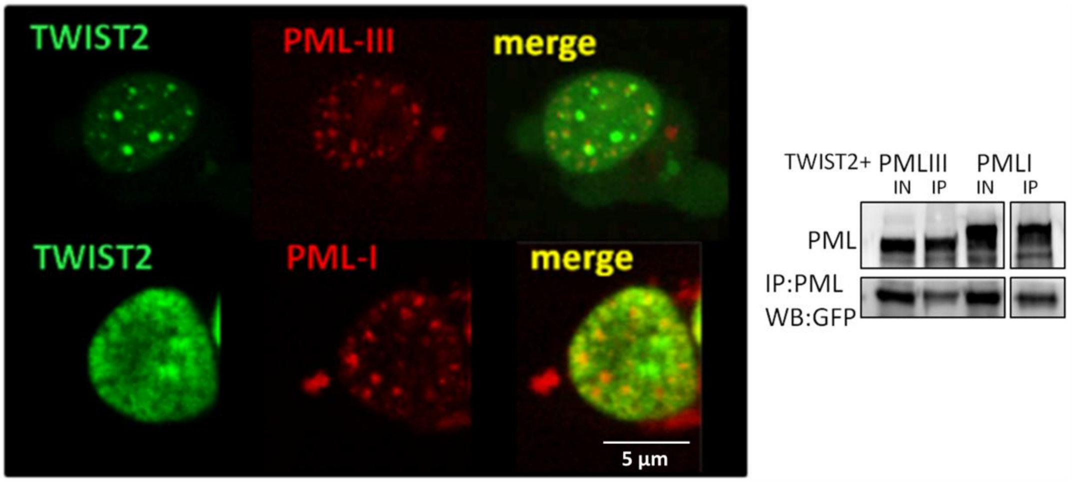

<u>Figure S4 (related to figure 3):</u> PML IV interacts specifically with bHLH domain of TWIST2. Left: Strong co-localization of PML IV (red) with shown TWIST2 deletions (green) by confocal microscopy. Scale bar 5µm. Middle: WB of PML, where specifically immunoprecipitates with the bHLH region of TWIST2 experiment following overexpression of PMLIV and TWIST2 –GFP fragments in HEK293T. Right: WB shows strong co-Immunoprecipitation of PML IV co-transfected with full length TWIST2-GFP in HEK293T cells. IgG control antibody is included to confirm specificity. Shown one out of at least two independent experiments with similar results.

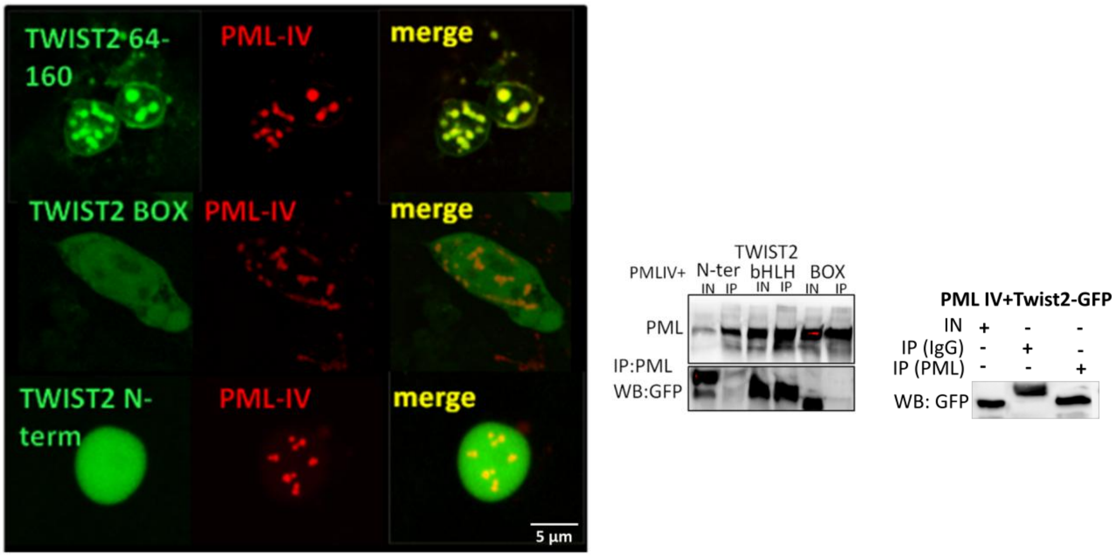

<u>Figure S5 (related to figure 4):</u> Bioinformatics analysis and validation of MCF7.

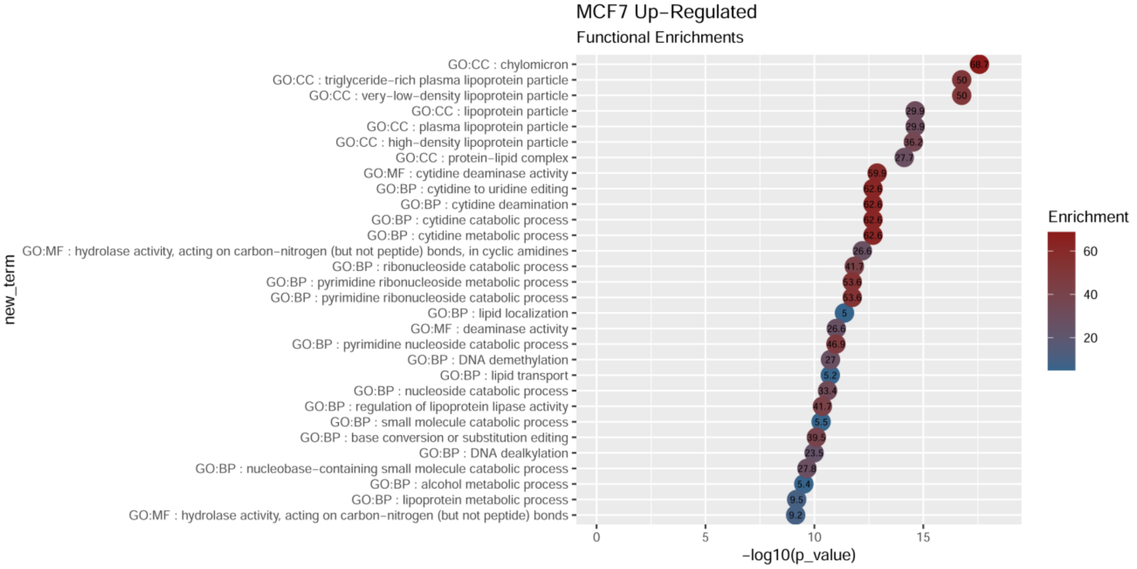

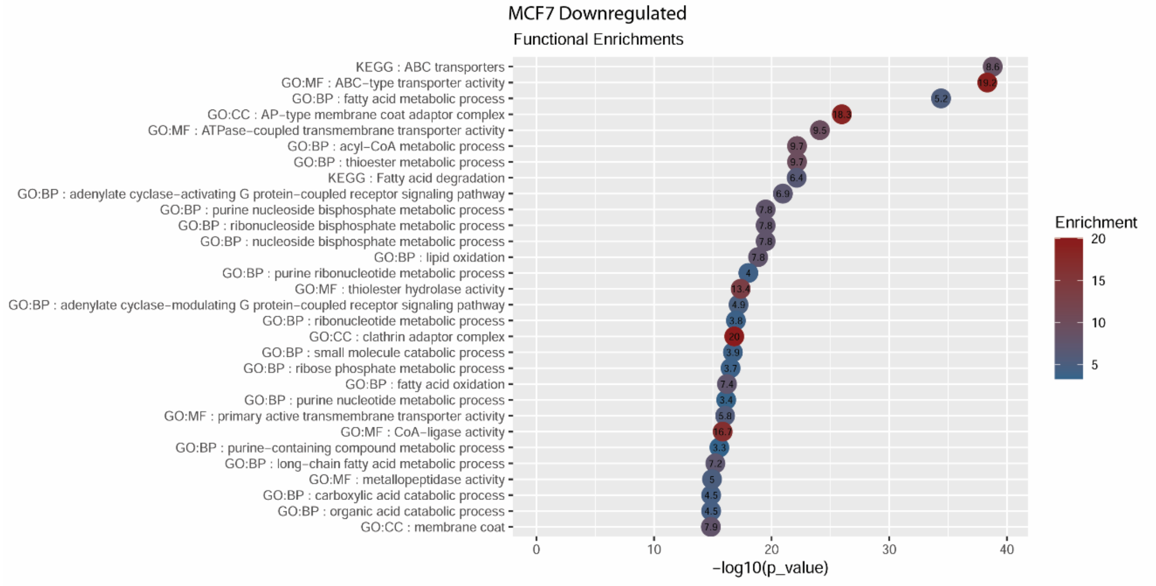

<u>MCF7 gene expression validations</u>

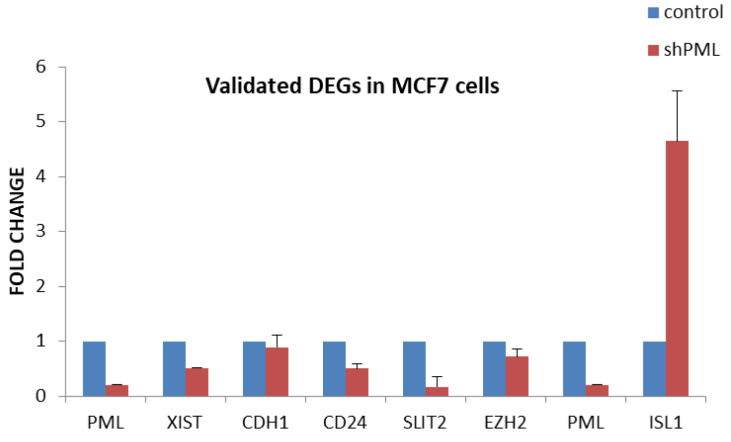

<u>Figure S6 (related to figure 6): MCF7 control and KD PML cells showed no metastatic lesions.</u>

Lungs from MCF7 control and PML-KD. (A) Examination of individual mouse control or PML-KD lungs by visible (upper panels) and fluorescence stereomicroscopy (lower panels). (B) Summary table of metastases in the MCF7 control and PML-KD groups. (C) Expression of PML protein in tumor xenografts and parental cell lines.

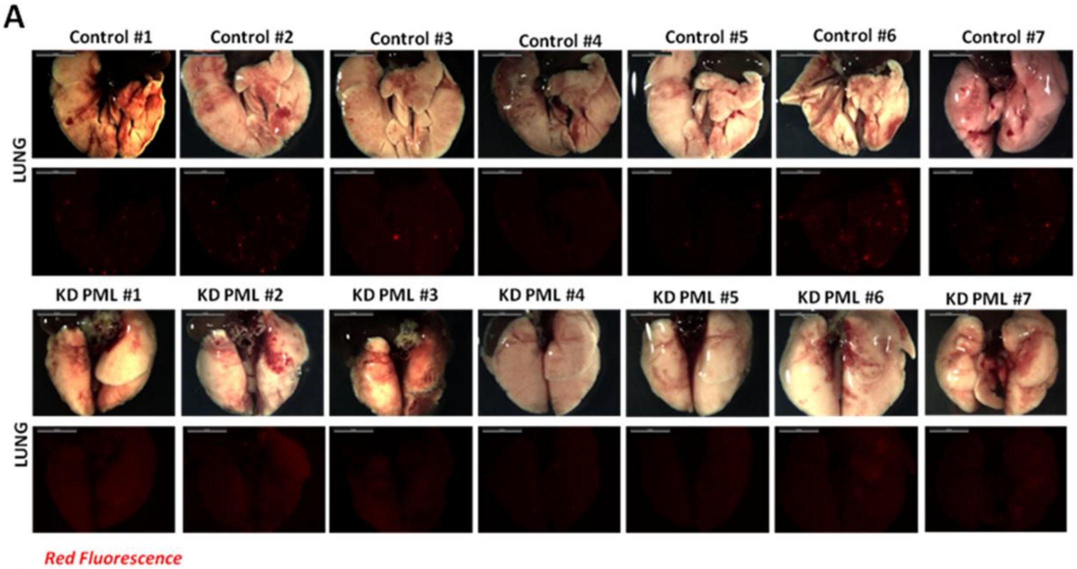

B

Organ Metastatic Intensity (Autopsy and Stereoscopic Fluorescence Observation)				
Cell type	MCF7			
	control		KD PML	
Lung	7/7	++	7/7	+
Liver	1/7	+	2/7	+
Axillary LN	0/7		1/7	+
Femoral Bone	0/7		0/7	

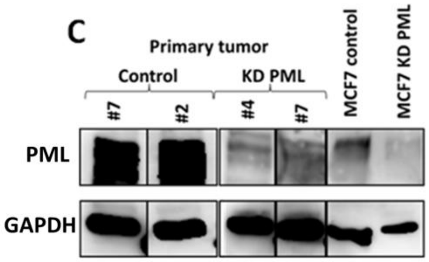

<u>Figure S7 (related to figure 6):</u> Xenograft derived cell lines maintained PML silencing and showed increased expression of highly aggressive metastasis–prone tumors related markers. (A) Left: Reduced PML RNA expression is maintained among parental lines and xenografts. Right: anti-PML staining shows the maintenance of PML-KD state in comparable control and PML–KD lung foci. (B) WB (right) and quantitation (left) of PML protein expression in primary tumor xenografts and their metastases. (C) mRNA expression of EpCAM and CD49f (ITGA6) in cell lines and primary tumors compared with control groups. Mean fold change ± SD from one triplicate.

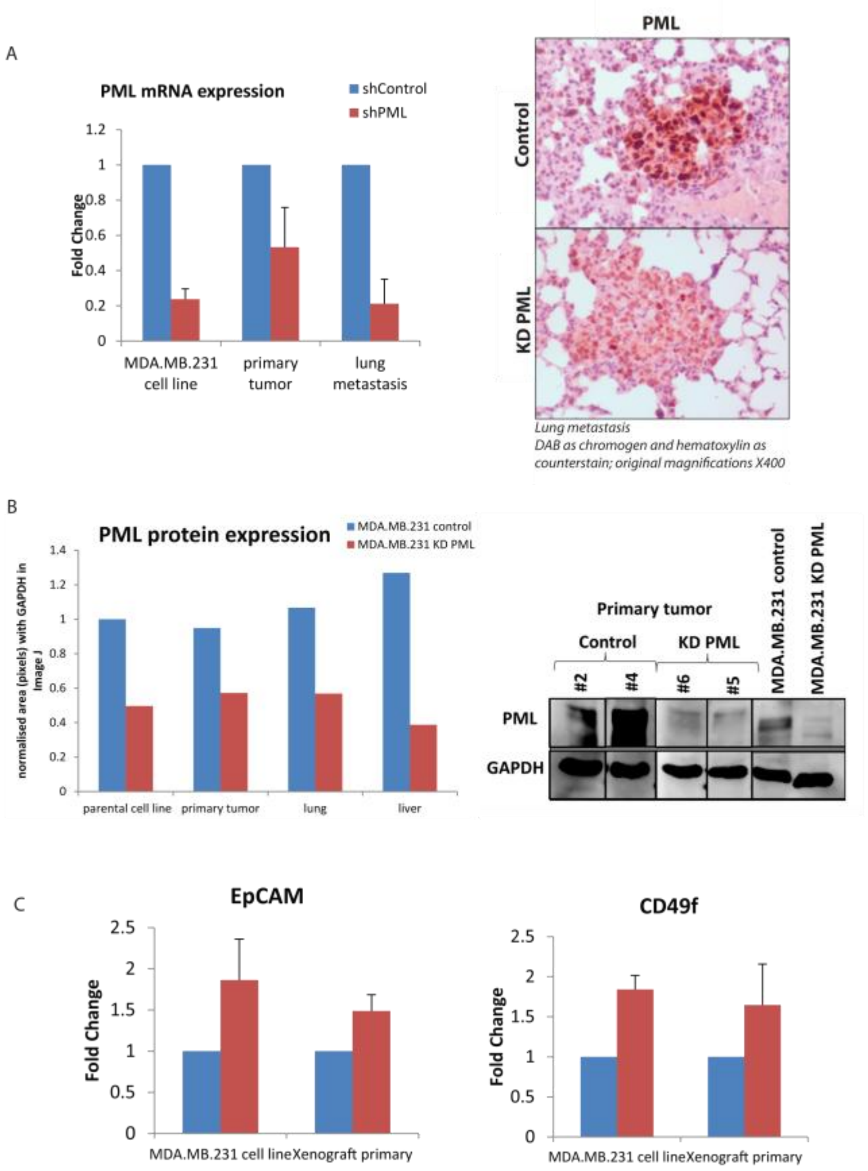

<u>Figure S8 (related to figure 6):</u> PML independently suppresses HIF1a and TWIST2 signaling. (A) Reduced expression of HIF1a and its target VEGFa gene in HIF1a-KD of control (deep & light blue) respectively or PML –KD MDA-MB-231 (red & pink respectively). (B) Expression of VEGFa and HIF1a gene in MDAMB231 line N2[11] without (N2) or after Doxycyclin inducible expression of PMLIV (N2+DOX). (C) Expression of CD24 and CDH1 gene in MDA-MB-231 control group (N2) and inducible expressing PMLIV group (N2+DOX). (D) Expression of CD24 and CDH1 genes in MDA-MB-231, shCONTROL or KD PML (deep blue & red respectively) or additional HIF1a KD in the above cell lines: shCONTROL/shHIF (light blue) and KD PML/shHIF (pink). Results show mean ±SD from at least 2 independent triplicates. * *t-test p-value≤ 0.05,* ***t-test p-value≤ 0.01,* ****t-test p-value≤ 0.001*

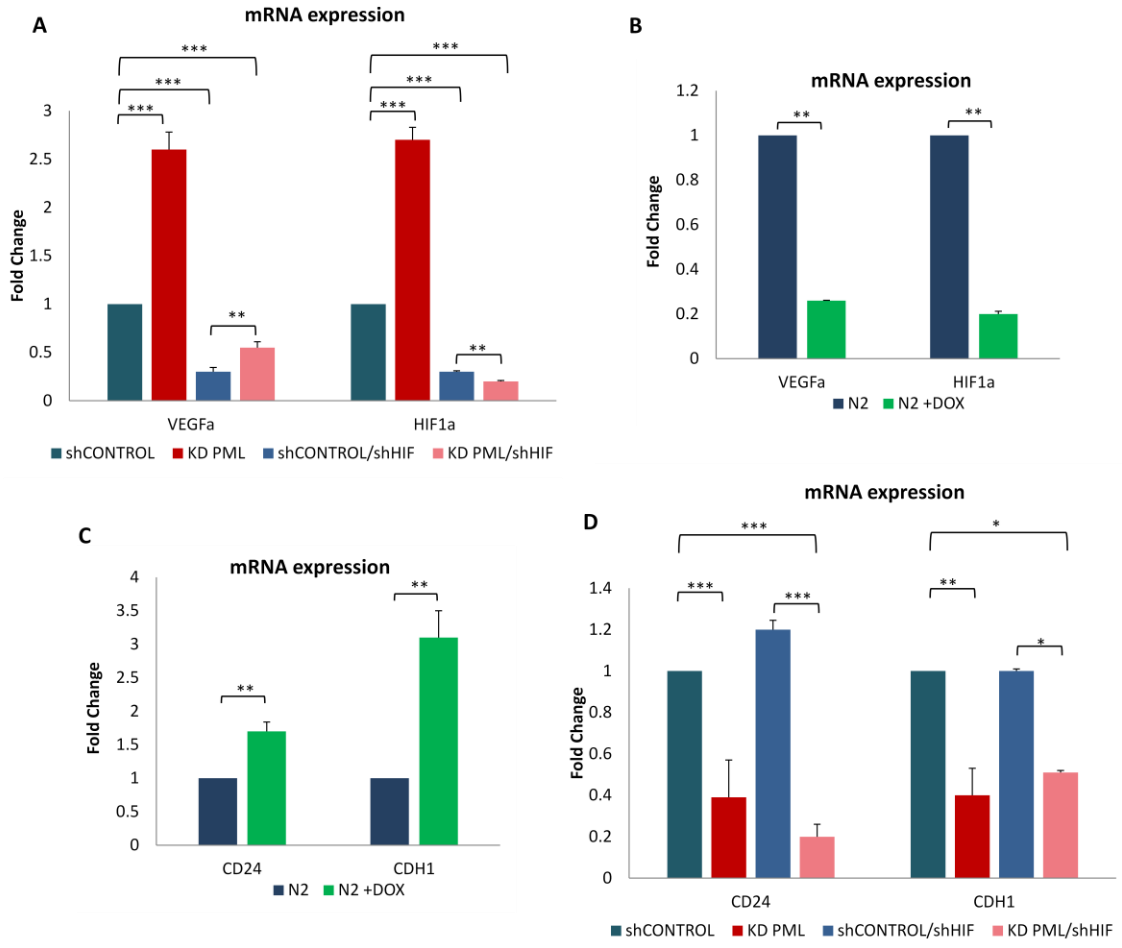

Supplement: Supplementary file 1 — Fig. S1. (related to Fig. 5): Increased in vivo tumor growth of MDA‐MB‐231 PML‐KD lines by two different shRNAs relative to controls. Fig. S2. (related to Fig. 2): PML loss enhances mesenchymal properties in MCF7 breast cancer cells. Fig. S3. (related to Fig. 3): PML I & III isoforms examined for interaction with TWIST2. Fig. S4. (related to Fig. 3): PML IV interacts specifically with the bHLH domain of TWIST2. Fig. S5. (related to Fig. 4): Bioinformatics analysis and validation of MCF7. Fig. S6. (related to Fig. 6): MCF7 control and KD PML cells showed no metastatic lesions. Fig. S7. (related to Fig. 6): Xenograft‐derived cell lines maintained PML silencing and showed increased expression of highly aggressive metastasis‐prone tumor‐related markers. Fig. S8. (related to Fig. 6): PML independently suppresses HIF1a and TWIST2 signaling. [file MOL2-17-2090-s001.pdf]
