# Supplementary material for: Promyelocytic leukemia protein regulates angiogenesis and epithelial–mesenchymal transition to limit metastasis in MDA‐MB‐231 breast cancer cells
Source: Mol Oncol. 2023 Sep 4;17(10):2090–108. doi: 10.1002/1878-0261.13501 (PMC10552902; doi:10.1002/1878-0261.13501)
Supplement: Supplementary file 3 — Table S3. (related to Fig. 7): List of 15 common genes between lung metastasis signature gene set [14] and MDA‐MB‐231 DEGs. [file MOL2-17-2090-s002.docx]

**Table S3 (related to figure 7): List of 15 common genes between Lung metastasis signature gene set [14] and MDAMB231 DEGs.**

| Gene name | Fold Change in MDAMB231 parental cell line DEGs | Fold change in Minn et al 2005 |
| --- | --- | --- |
| C4BPB | 0.460635 | 0.16 |
| CASP1 | 4.5 | 3.54 |
| EDIL3 | 2.25228 | 0.24 |
| EMP1 | 2.10141 | 3.15 |
| EPHX1 | 3.333333 | 0.30 |
| EREG | 3.197861 | 12.82 |
| FSCN1 | 0.407867 | 3.99 |
| ID1 | 0.30254 | 3.12 |
| KIAA1199 | 0.280992 | 0.07 |
| MBNL2 | 1.988971 | 0.33 |
| MMP1 | 9.014286 | 13.35 |
| PTGS2 | 9.625 | 6.23 |
| RARRES3 | 0.232759 | 0.22 |
| SPINK4 | 0.237762 | 0.16 |
| TNC | 3.416667 | 8.99 |

[14] Minn AJ, Gupta GP, Siegel PM, Bos PD, Shu W, Giri DD, et al. Genes that mediate breast cancer metastasis to lung. *Nature*. 2005;**436**(7050):518–524.
